# Exploratory Study of Executive Function Abilities Across the Adult Lifespan in Individuals Receiving an ASD Diagnosis in Adulthood

**DOI:** 10.1007/s10803-018-3675-x

**Published:** 2018-07-06

**Authors:** Patricia Abbott, Francesca G. Happé, Rebecca A. Charlton

**Affiliations:** 1 Autism Diagnostic Research Centre, Southampton, UK; 2 Social, Genetic and Developmental Psychiatry Research Centre, Institute of Psychiatry, Psychology and Neuroscience, King’s College London, London, UK; 3 Department of Psychology, Goldsmiths University of London, New Cross, London, SE14 6NW UK

**Keywords:** Ageing, Autism spectrum disorders, Cognition, Executive function, Lifespan

## Abstract

Little is known about cognition in autism spectrum disorder (ASD) across adulthood. We examined executive function abilities and autism traits in 134 adults receiving a first diagnosis of ASD. Participants aged 18–75 years with abilities in the normal range were assessed on executive function and self-report autism traits. Results suggest that for some abilities relying on speed and sequencing (Trails A and B; Digit Symbol), late-diagnosed individuals with ASD may demonstrate better performance than typical age-norms. On other executive measures (Digit Span, Hayling and Brixton tests) age-related correlations were similar to typical age-norms. Different domains of executive function may demonstrate different trajectories for ageing with ASD, with patterns of slower, accelerated or equivalent age-related change being observed across different measures.

## Introduction

Cognitive changes are common across the adult lifespan with a general decline in abilities from early adulthood onwards, but we know relatively little about the adult lifespan in autism spectrum disorder (ASD). With ASD being formally added to the International Classification of Diseases (ICD) version 9 in 1977 (World Health Organisation 1977) and the Diagnostic and Statistical Manual of Mental Disorders (DSM) version III in 1980 (American Psychiatric Association 1980), individuals diagnosed with ASD in childhood are only now in mid-life. Although individuals diagnosed earlier may have received a diagnosis of Childhood Schizophrenia, differences in diagnostic criteria over time and the increase in familiarity with ASD are likely to introduce bias and extraneous variability into cross-sectional investigations across the lifespan in ASD (King and Bearman 2009; Rutter 2005). In recent years receiving a first diagnosis of ASD in adulthood or even later life has become more common (Geurts and Jansen 2012; Heijnen-Kohl and van Alphen 2009; Jones et al. 2014). Individuals receiving a “late” diagnosis are unlikely to represent the whole spectrum of ASD, as they often have abilities in the normal range and have not previously come to the attention of clinical services. However individuals receiving a diagnosis of ASD according to current criteria, provide a unique opportunity to explore cognitive abilities across the lifespan. When comparing adults with ASD diagnosed in childhood, there is a risk of a confounding chronological age and changes in diagnostic criteria overtime. In contrast comparing adults of different ages receiving a first diagnosis according to the same, current criteria, has some advantages. These individuals allow us to begin to examine the adult lifespan and age-related associations in ASD, albeit with caveats about generalisability. Examining cross-sectional age-associations in individuals diagnosed according to the same criteria will indicate where future longitudinal studies should focus. In this paper we describe executive function abilities in adults receiving a diagnosis of ASD, and explore effects associated with age, comorbidities and ASD symptoms.

ASD and typical ageing are both associated with cognitive difficulties in the domains of executive function (Amieva et al. 2003; Hill 2004). Children with ASD demonstrate lower performance on measures of spontaneous and reactive flexibility, inhibitory control, planning, and working memory compared to age and IQ matched typically developing children (O’Hearn et al. 2008; Rosenthal et al. 2013). In early adulthood, ASD individuals who have IQ in the average range, continue to demonstrate reduced performance in some executive function domains compared to age and ability matched neurotypical adults (Wallace et al. 2016). It has been suggested that tasks requiring executive control or that draw on multiple aspects of executive function (i.e. towers tests, Wisconsin Card Sorting Test, spatial working memory), demonstrate more consistent difficulties among individuals with ASD compared to other measures (Hill 2004; Kenworthy et al. 2008). However, even within the domain of executive control, inconsistent results have been noted, likely influenced by differences in age and IQ across studies, or sampling and other methodological issues (Wilson et al. 2014). Furthermore relatively little is known about gender differences in executive function in adults with ASD or how these may be impacted across adulthood. A recent review identified gender differences in some (Trail-Making Test; Stop Test) but not all (Towers Test; Go-No-Go Test; Wisconsin Card Sorting Test) executive function measures in ASD adults (Hull et al. 2016).

Across the typical adult lifespan, age-related declines are observed in executive functions but the age at which decline begins and the trajectory of the decline varies across domains (Amieva et al. 2003; Bryan and Luszcz 2000; West et al. 2010). Research has suggested that tasks requiring reactive flexibility (including inhibitory control) are particularly affected across adult ageing (Madden et al. 2010). Other aspects of executive function also demonstrate declines from early adulthood (mid-twenties) onwards (Baltes and Lindenberger 1997) including spontaneous flexibility as often measured by verbal fluency (Hasher et al. 1999), planning (Amieva et al. 2003) and working memory (Charlton et al. 2010). The executive function difficulties observed in both young adults with ASD and across the typical adult lifespan have led to the suggestion that ageing with ASD may represent an additional risk (or double hit) for age-related cognitive decline (Happe and Charlton 2012; Lever and Geurts 2015). However, to date there is little evidence to support or refute this notion.

A number of possible trajectories for cognitive ageing with ASD have been hypothesised (Geurts and Vissers 2012; Happe and Charlton 2012; Lever and Geurts 2015). As stated above, the common difficulties in executive function noted in ASD and ageing may lead to older ASD individuals being at greater risk for cognitive decline than typical ageing peers. Alternatively the different processing style and altered brain networks observed in ASD at young ages (Happe and Frith 2006; Koshino et al. 2008; Wallace et al. 2015), may be protective against age-related change for individuals with ASD. Perhaps the alternative strategies for task completion, development of coping strategies and reliance on different brain networks often noted in ASD, protect individuals with ASD from the neurotypical age-related EF declines.

Although a number of studies examining cognition in ASD have included adults in mid-life or early old age (Bramham et al. 2009; Nyden et al. 2010; Spek et al. 2011; Ambery et al. 2006) few have directly examined age-effects. To our knowledge seven published studies to date have examined age associations in executive functions including “late” diagnosed adults with ASD in mid- or late-life (Braden et al. 2017; Davids et al. 2016; Geurts and Vissers 2012; Lever and Geurts 2015; Lever et al. 2015, 2017; Powell et al. 2017). One paper focused on working memory, and suggested that age-related working memory declines may be less steep in ASD than in typical ageing (Lever et al. 2015). However, a smaller study in middle age did not explore age-associations and demonstrated no group differences between ASD and matched neurotypical controls (Braden et al. 2017). Studies examining the executive functions of planning and spontaneous flexibility demonstrate inconsistent results but generally show few differences between ASD and typical adults in older populations (Davids et al. 2016; Geurts and Vissers 2012; Lever & Geurts, 2015). Performance on planning tasks (Towers tests), which have previously demonstrated poorer performance in young adults with ASD compared to typical adults, did not reveal group differences in accuracy with equivalent performance in ASD and typical peers in late-adulthood (Davids et al. 2016; Geurts and Vissers 2012). Planning performance was not associated with age for ASD or typical adults in either study. On spontaneous flexibility tests (measured by verbal fluency), performance by individuals with ASD was lower than age and IQ matched controls in two studies (Geurts and Vissers 2012; Lever and Geurts 2015) but not a third (Davids et al. 2016). Different associations with age were also observed. Results demonstrated typical age-related decline in semantic fluency for ASD individuals (Davids et al. 2016), no (expected) age-related declines in phonemic fluency in ASD or typical adults (Lever and Geurts 2015), and expected age-related decline in phonemic fluency for typical adults but not individuals with ASD (Geurts and Vissers 2012). Other studies measuring different aspects of flexibility demonstrate different patterns of results. Cognitive flexibility measured by the trail making test switching subscale, demonstrated a steeper age-association (poorer performance with older age) in ASD compared to matched controls (Powell et al. 2017). By contrast, a Simon Task used to calculate both Reactive and Spontaneous Control, found no differences between ASD and typical controls in terms of associations with age (Lever et al. 2017). In a recent small study examining executive function in middle-aged men with ASD compared to age-matched controls, individuals with ASD made more errors on the Wisconsin Card Sorting Test (generally considered to measure perseveration), but age effects were not measured (Braden et al. 2017). Although there are differences between the samples described in these papers, there are no characteristics that obviously account for the pattern of results. For all papers, the ASD group have similar IQ (all > 80; Mean ≈ 110). Two of the studies include only adults aged over 50 years of age (Davids et al. 2016; Geurts and Vissers 2012) and report very similar scores for the ASD group on the Social Responsiveness Scale (Davids et al., t-score = 64; Geurts and Vissers, t-score = 69). Four of the remaining papers include adults from a wider age range including young adults, with the three papers by Lever and colleagues reporting on largely the same participants (aged 19–79 years; Powell et al. aged 30–67 years).

Different domains may be differentially affected across adulthood in ASD and show different trajectories of increased or decreased risk in age-related associations. Even across three studies that examined the same ASD population, different aspects of executive function demonstrated different age-associations ; working memory and semantic fluency showed no expected age-related decline (Geurts and Vissers 2012; Lever et al. 2015), but phonemic fluency, reactive and proactive control showed the same age-related decrement in ASD as in typical adults (Lever et al. 2017; Lever and Geurts 2015). It has been suggested that different aspects of cognition may underlie different symptoms/behavioural features in ASD (Brunsdon and Happe 2013; Happe and Ronald 2008), in which case cognition-symptom associations may be informative, but this has not yet been examined across the lifespan.

Individuals with ASD also demonstrate high levels of anxiety and depression compared to typical adults, which may interact with cognitive function (Ghaziuddin et al. 2002; Lai et al. 2011; Lever and Geurts 2016). Few previous studies report the presence of common comorbid difficulties such as anxiety and depression or medications associated with treatment of these difficulties. Such difficulties (as well as comorbid epilepsy, intellectual ability, socioeconomic status, etc) are known to affect executive function performance and could explain some of the variability in findings around executive function, age and ASD. In one study examining informant-reported executive function and mood in young adults with ASD, associations between mood and cognition were observed independent of IQ (Wallace et al. 2016; flexibility with anxiety, metacognition with depression). To our knowledge only one study to date has examined mood disorders in ageing with ASD (Lever and Geurts 2016). Among individuals with ASD 79% had ever met criteria for a psychiatric disorder, compared to the population rate of 40% (Lever and Geurts 2016; Kessler et al. 2005). Across the adult lifespan (19–79 years), age was not associated with levels of anxiety or depression. Among individuals with ASD, the prevalence of mood and anxiety disorders were lower in older adults compared to young and middle-aged adults, but results did not reach significance for every specific disorder (Lever and Geurts 2016). Among typical older adults, although levels of depression and sub-clinical low mood are common (Barch et al. 2012; Beekman et al. 1999) some studies have suggested lower anxiety levels (Jorm 2000), and improved mood and well-being with age (Reed and Carstensen 2012; Carstensen et al. 2011). Thus, if individuals with ASD show the same pattern of reduced symptoms of anxiety and depression in ageing, then there may be beneficial effects on well-being and cognition for older ASD adults.

In this study we examined executive functions in a group of adults receiving a first diagnosis of ASD at a specialist ASD diagnostic centre. For age-cognition associations, if the increased risk hypothesis is correct then one would predict a stronger negative correlation between age and executive function measures in the ASD group compared to normative data for age-matched typical adults. If, however, the protective hypothesis is correct, then one would expect a significant positive correlation of EF with age in ASD—due to younger adults having average or poor standardised scores and older ASD adults having higher standardised scores. Of course an alternative is equivalent performance to normative data for age-matched typical adults, reflected in a non-significant correlation between age and cognitive function measured using age-normative data and standardised scores. Given the paucity of published studies, we do not have a clear prediction about which of these hypotheses will be supported, and this study is therefore exploratory in nature. However we expect that even within the domain of executive function the pattern of age-related association will differ across tasks with some showing less age-related change in ASD than in typical adults, others more or equivalent change. In terms of the predicted associations between age and autism traits, previous studies have suggest both linear and non-linear associations with age (Happe et al. 2016; Lever and Geurts 2018); and we predict that age will be associated with more severe traits. Previous studies have reported associations between ASD traits and executive function (Brunsdon and Happe 2013; Happe and Ronald 2008), but this has not been explored across adulthood.

## Methods

### Participants

The Autism Diagnostic Research Centre (ADRC) received referrals to their adult service for individuals over 18 years of age with IQ in the normal range. Referrals were typically through General Practitioner surgeries (in the UK all individuals are registered with a family doctor General Practitioner surgery), or Autism Oxford following a pre-assessment interview. ADRC was not a service for individuals with a learning disability: although adults with a learning disability are sometimes referred to the ADRC they are typically referred to other services prior to initial appointment. When an intellectual or learning disability is suspected during assessment at ADRC, IQ is measured and individuals are referred on to appropriate services. Therefore the individuals included here were all considered to have IQ in the normal range (IQ ≥ 80).

All 454 adults referred to the ADRC service between March 2008 and May 2016 were asked at the time of the assessment whether de-identified data from their assessment could be used for research purposes. Please note, not all individuals received a diagnosis. 167 adults who subsequently received a diagnosis of ASD gave written informed consent for data to be used for research purposes. Additional ethical approval for use of this existing data was awarded by the Goldsmiths University of London Ethics Committee, and all research was carried out according to the Declaration of Helsinki. Of the 167 individuals with ASD who provided consent for their information to be used, six were excluded due to being aged under 18 years old, and eight were excluded as they were found to have a learning disability and were referred to other services. Of the remaining 153 adults with ASD, 19 did not have data available on all eight neuropsychological measures examined in the present analysis. Therefore, 134 adults (males = 97; females = 37) with complete data will be described here. Details of participants are presented in Table 1 including age distribution.

**Table 1:** Demographic information for males and females receiving a diagnosis of ASD

	Male N = 97	Female N = 37	Statistics
Age (mean, SD) Range N by age-ranges	31.38 (11.99) 18–74 (18–29, n = 51; 30–39, n = 23; 40–59, n = 20; >60, n = 3)	30.51 (11.97) 18–75 (18–29, n = 20; 30–39, n = 12; 40–59, n = 4; >60, n = 1)	F = .140, p = .708
Highest educational level^a^ (%)	No qualifications = 7.2% GCSE level = 30.9% Post-16 qualifications = 4.1% A-level = 25.8% Diploma = 3.1% Degree = 8.2% Post-graduate = 4.2% (missing = 16.5%)	No qualifications = 2.7% GCSE level = 16.2% Post-16 qualifications = 2.7% A-level = 40.5% Diploma = 0% Degree = 21.6% Post-graduate = 2.7% (missing = 13.5%)	*X* ^2^ = 10.94, p = .141
In employment/education (yes, no, retired)	49.4, 40.2%, 3.1% (missing 7.2%)	48.6%, 40.5%, 0% (missing 10.8%)	*X* ^2^ = .637, p = .425 (retirees omitted)
Employment details	None = 9.3% None-unable to cope = 6.2% Voluntary work = 4.1% Working or seeking work = 52.5% Studying = 17.5% Retired = 3.1% (missing = 7.2%)	None = 13.5% None-unable to cope = 10.8% Voluntary work = 5.4% Working or seeking work = 35.1% Studying = 24.3% Retired = 0% (missing = 10.8%)	–
Family history of ASD (yes-diagnosed, no)	5.92	5.32	*X* ^2^ = 2.71, p = .138
Family history	None = 81.4% Family diagnosed = 5.2% Family suspected = 3.1% Other Dev/Psych = 10.3%	None = 75.7% Family diagnosed = 13.5% Family suspected = 2.7% Other Dev/Psych = 8.1%	–

^a^For whole sample: no qualifications = 6%; GCSE level = 26.9%; Post-16 qualifications = 3.7%; A-level = 29.9%; Diploma = 2.2%; Degree = 11.9%; Post-graduate = 3.7% (missing = 14.9%). UK population average: no qualifications = 22.5; GCSE up to 1 A-level = 28.5%; 2 or more A-level = 12.4%; Undergraduate degree or higher = 27.4%

### Assessment

The diagnostic process at ADRC included interviews and assessment by an expert team of clinical psychologists, neuropsychologists and psychiatrists with the assessment personalised to each individual. The assessment typically lasted a whole day and included prior completion of self-report questionnaires, recording of demographic information, structured clinical interviews, neuropsychological assessment, and observations of interactions in informal settings (e.g., during lunch). Diagnosis according to ICD-10 criteria (F84: Pervasive Developmental Disorders; F84.0 Childhood Autism; F84.1 Atypical Autism; F84.5 Asperger’s Syndrome) was made by the clinical team based on the assessment. This paper will focus on data from the neuropsychological assessment, specifically examining executive function, but will also consider responses to self-report questionnaires.

Demographic information was recorded through self-report and included age, sex, highest education level, employment history, and any family history of developmental or psychiatric disorders. Prior to the assessment, individuals were asked to complete three self-report questionnaires examining ASD traits, the Autism Quotient (AQ; Baron-Cohen et al. 2001), the Empathy Quotient (EQ; Baron-Cohen and Wheelwright 2004), and the Systemising Quotient (SQ; Baron-Cohen et al. 2003). One individual did not complete any ASD trait questionnaires, and one additional individual did not complete the SQ. On the day of assessment, some (but not all) individuals were also asked to complete the Beck Depression Inventory II (BDI; Beck et al. 1996) and Beck Anxiety Inventory (BAI; Beck et al. 1988). Note, as appointments constituted a clinical assessment, measures not considered relevant to the individual were not administered. Depression and anxiety ratings are available for 73 individuals (males = 51; females = 21). Note, no differences in demographic variables, ASD traits or mean EF were noted between those who did or did not complete the mood questionnaires.

### Neuropsychological Assessment

A core battery of standardised neuropsychological assessments was routinely performed for all individuals, however occasionally measures were omitted due to time constraints. All the standardised neuropsychological measures have normative data for typical adults across different specific age-bands, enabling comparison of individual data to age-specific population norms. The battery included detailed assessment of different aspects of executive function including: the Digit Symbol subtest (executive control) from the Wechsler Adult Intelligence Scale III or IV (WAIS; Wechsler 1997, 2008), Digit Span subtest (working memory) from the WAIS represented as age-scaled scores, the Hayling (initiation; executive control) and the Brixton (reasoning) tests (Burgess and Shallice 1997) represented as scaled scores, Trails A and B (sequencing; executive control) time to complete (Army Individual Test Battery 1944) with raw scores converted to age-corrected percentile scores (Davies 1968 in; Mitrushina et al. 1999), and Zoo Map (planning) and Key Search (planning) subtests from the Behavioural Assessment of the Dysexecutive Syndrome (Wilson et al. 1996) recorded as profile scores. Although IQ scores were not available, mean scores from WAIS subtests indicate that the participants have abilities in the average range (Digit Symbol, Mean = 9.23; Digit Span, Mean = 9.66).

A composite score for executive function was computed (Mean EF) for use in the analysis in order to measure EF as a domain (alongside measuring sub-domains of EF). Cronbach’s alpha was calculated to assure that all variables measured an underlying construct; internal consistency was good (alpha = .75) and the alpha was not substantially improved by excluding any measure. As each measure was recorded on a different scale, individual measures were recoded as percentile scores. Percentile scores were selected as two tests were already represented as percentile scores and clear conversion to percentiles were available for another four tests. Specifically, Digit Symbol and Digit Span subtests were recoded based on Sattler (Sattler 1988 in; Spreen and Strauss 1998); the Hayling and Brixton tests were recoded using conversion criteria in the test manual (Burgess and Shallice 1997); Zoo Map and Key Search were recoded using the mean and standard deviations reported in the manual (Wilson et al. 1996), based on 0.3 of a SD being equivalent to 13 percentile points; Trails A and B were already represented as percentile scores and were not recoded. The mean of the eight percentile scores created the composite EF score (Mean EF).

### Statistical Analysis

Differences between male and female individuals receiving a diagnosis of ASD were examined using ANOVA (for continuous variables) and Chi square (for categorical variables) on demographic information, ASD traits, mood and cognitive function. Associations between variables of interest and age were examined using Pearson’s correlation coefficient. Linear Regression was utilised to examine the variables that explained variance in EF performance and the cognitive variables associated with ASD traits measured by self-rated AQ scores. Analyses explored any confounds of age or psychiatric symptoms with gender, in exploring age—EF associations. Due to the exploratory nature of this study results were not corrected for multiple comparisons.

## Results

Participants were 134 adults (males = 97; females = 37). The age range for those receiving a diagnosis of ASD as adults was between 18 and 75 years old (mean = 31.14, SD = 11.94). The majority of participants were < 60 years old, with only four individuals being older than this (1. male, 63 years old, UK GCSE/16-year old level qualifications; 2. male, 67 years old, education data missing; 3. male, 74 years old, undergraduate degree; 4. female, 75 years old, UK A-level/18 year old level qualifications). To assure that results are not due to the influence of these few older adults, some analyses are repeated excluding these four individuals.

Highest level of education was available for 113 of the 134 participants [missing data for males, n = 16 (16.5%) and females, n = 5 (13.5%)]. Full details of education level is provided in Table 1. It is worth noting that only eight individuals (7.1%, seven males; one female) reported having no qualifications compared to the UK population average of 22.5% (Office for National Statistics, National Records of Scotland, and Northern Ireland Statistics and Research Agency, 2016). The range of educational attainment in this sample includes all levels from the UK exams at age 16 (n = 36, 31.9%), through to postgraduate degrees (n = 5, 4.5%; 2 Masters, 3 PhD).

### Gender Differences

No significant differences between males and females were observed on any demographic variable, see Table 1. No significant gender differences were observed on self-report ASD traits or mood scales, see Table 2.

**Table 2:** Self-report ASD traits and mood scales by gender

	Male N = 96^a^	Female N = 37	Statistics
AQ (mean, SD) Range	34.04 (7.55) 13–48	36.59 (6.88) 19–48	F(1,131) = 3.20, p = .076 Cohen’s *d* = .35
EQ (mean, SD) Range	20.71 (10.31) 1–50	19.95 (10.44) 5–42	F(1,131) = .145, p = .704 Cohen’s *d* = .07
SQ (mean, SD) Range	60.93 (26.66) 0-129	61.95 (25.33) 13–118	F(1,130) = .040, p = .842 Cohen’s *d* = .03

	N = 51	N = 22	
BDI (mean, SD) Range	18.94 (13.43) 0–53	20.64 (15.86) 0–49	F(1,71) = .219, p = .641 Cohen’s *d* = .12
BAI (mean, SD) Range	15.06 (12.49) 0–52	20.59 (14.70) 0–50	F(1,71) = 2.71, p = .104 Cohen’s *d* = .41

^a^For the SQ n = 95

Mean scores by gender are reported in Table 3; note that mean scores for males and females are within the average range based on standard scores for each executive function measure. For measures of executive function, females scored significantly higher than males on the Digit Symbol test, no other gender differences reached significance.

**Table 3:** Mean (and standard deviations) for executive function tasks by gender

	Male N = 97	Female N = 37	Statistics
Digit symbol scaled score^a^	8.04 (3.01)	10.41 (3.20)	**F(1,132) = 15.93, p **< **.001** **Cohen’s** ***d*** =** .76**
Digit span scaled score^a^	9.72 (3.51)	9.59 (2.85)	F(1,132) = .039, p = .844 Cohen’s *d* = .04
Brixton-scaled score^b^	6.85 (1.99)	6.35 (1.96)	F(1,132) = 1.66, p = .200 Cohen’s *d* = .25
Hayling total-scaled score^b^	5.41 (1.45)	5.46 (1.59)	F(1,132) = .027, p = .870 Cohen’s *d* = .03
Trial A new percentile^c^	55.36 (32.98)	64.59 (30.63)	F(1,132) = 2.18, p = .142 Cohen’s *d* = .29
Trails B new percentile^c^	60.21 (28.57)	64.05 (28.50)	F(1,132) = .487, p = .487 Cohen’s *d* = .14
Key search profile score^d^	2.57 (1.29)	3.03 (1.19)	F(1,132) = 3.55, p = .062 Cohen’s *d* = .37
Zoo map profile score^d^	2.43 (1.18)	2.43 (.867)	F(1,132) = .000, p = .998 Cohen’s *d* = 0
Mean EF percentile^c^	52.31 (17.48)	58.11(16.82)	F(1,132) = 3.01, p = .085 Cohen’s *d* = .34

Significant result is given in bold

^a^Range = 1–20, average = 10

^b^Range = 2–10, average = 6

^c^Range = 1–100, average = 50

^d^Range = 0–4, average = 2

Since there were few gender differences on these measures, subsequent analyses were performed on the whole sample with gender being examined only in measures demonstrating group differences.

### Correlations with Age

Note that analyses are performed on age-scaled scores, therefore if performance is in keeping with typical ageing then no age effects would be observed.

**Fig. 1:**
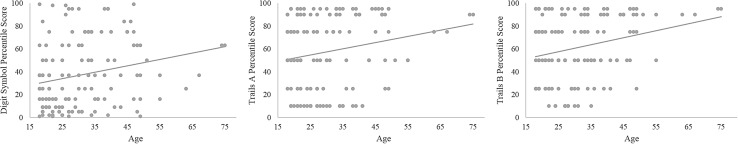
Scatterplots showing correlations between age and Digit Symbol, Trails A and Trails B

Results demonstrate that age correlates significantly with performance on Digit Symbol, Trails A and B, indicating higher scores with older age (see Table 4; Fig. 1 for details). As gender differences were observed for Digit Symbol, age-correlations were examined by gender for this task. Correlations were similar for males and females although neither reached significance in the gender sub-samples. When the EF composite score was examined, no significant correlation with age was observed.

**Table 4:** Correlations with age

	Whole sample (n = 134)	Sample excluding adults > 60 years old (n = 130)
EF measures (n = 134)		
Digit symbol scaled score^a^	**r** = .**204, p** = .**018**	**r** = .**226, p** = .**010**
Digit span	r = .101, p = .244	r = .072, p = .417
Brixton-scaled score	r = − .127, p = .142	**r** = **− .205, p** = .**019**
Hayling total-scaled score	r = − .105, p = .226	r = − .086, p = .329
Trial A new percentile	**r** = .**201, p** = .**020**	r = .151, p = .086
Trails B new percentiles	**r** = .**257, p** = .**003**	**r** = .**182, p** = .**038**
Key search profile score^a^	r = .121, p = .163	r = .047, p = .598
Zoo Map profile score	r = − .153, p = .078 Cohen’s *d* = .31	**r = − .224, p** = .**010**
Mean EF percentile^a^	r = .127, p = .145	r = .046, p = .604
AQ (n = 133)^a^	**r** = .**219, p** = .**012**	**r** = .**256, p** = .**003**
EQ (n = 133)	r = − .003, p = .973	r = .028, p = .749
SQ (n = 132)	**r** = .**333, p** < .**001**	**r** = .**366, p** < .**001**
BDI (n = 73)	r = − .138, p = .246	r = − .144, p = .235
BAI (n = 73)	r = − .208, p = .077 Cohen’s *d* = .43	r = − .165, p = .161

Significant results are given in bold

^a^Correlations for males and females provided where group differences are significant or showing a trend towards significance for gender differences. Digit Symbol: male r = .217, p = .033, female r = .253, p = .132; key search: male r = .112, p = .273, female r = .175, p = .301; mean EF: male r = .101, p = .324, female r = .222, p = .187; AQ: male r = .264, p = .009; female r = .121, p = .477. NB correlations are not significantly different to each other on Fisher’s r: Digit Symbol scaled score (z = − .19, p = .849); Key Search Profile Score (z = − .32, p = .749); Mean EF percentile (z = − .62, p = .535); AQ (z = .74, p = .459)

When these analyses were repeated omitting the four older adults (n = 130), the correlations between age and Digit Symbol and Trails B remained significant and age-correlations with the Brixton and Zoo Map tests reached significance (see Table 4). The association between age and Trails A no longer reached significance.

Self-report measures of ASD traits were examined for associations with age. AQ and SQ scores both correlated significantly with age, with higher scores associated with older age. EQ scores did not correlate with age. (Note, none of the ASD trait questionnaires are age-standardised.) A non-significant correlation with age was observed for the BAI (medium effect size), where lower anxiety scores were associated with older age. BDI scores did not correlate with age. Omitting the four older adults and repeating these analyses produced the same pattern of results (see Table 4).

Analyses were repeated to examine non-linear effects but did not improve the models, therefore the results are not reported.

### Correlations Between EF and Self-report ASD Traits

**Fig. 2:**
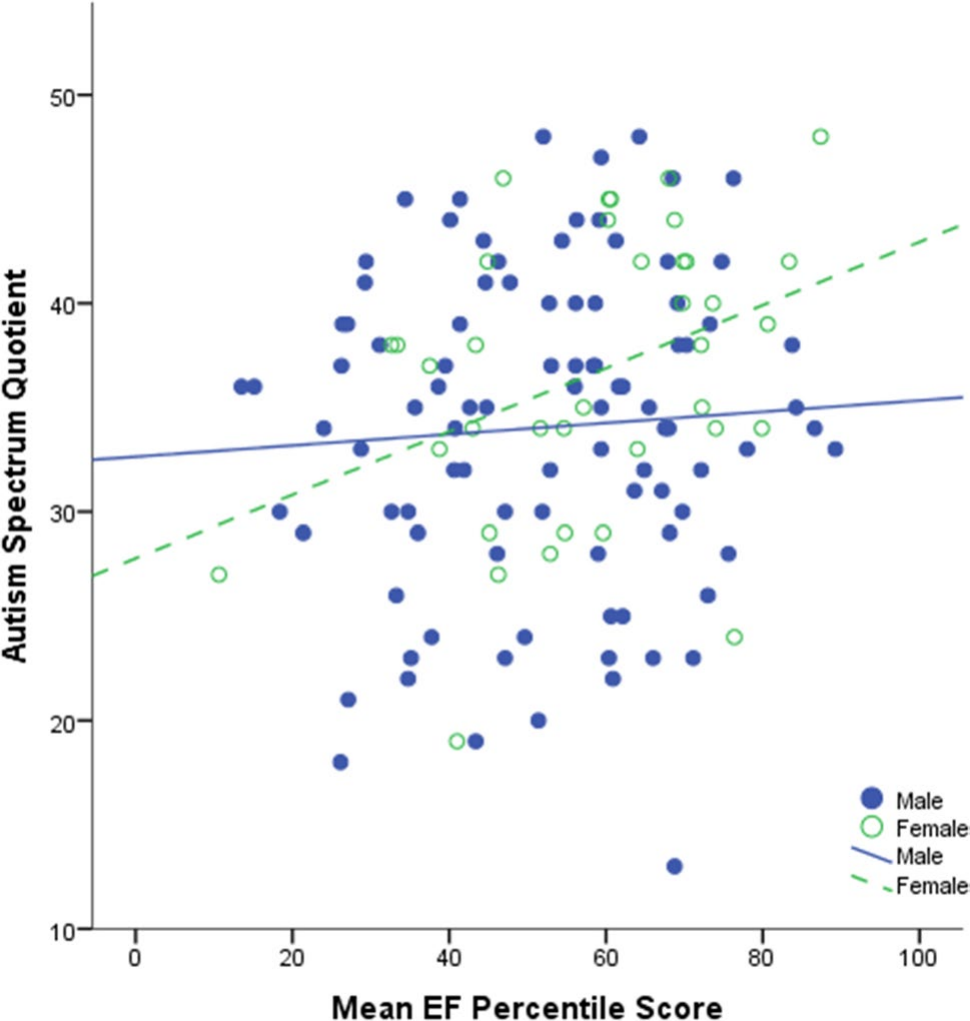
Correlation between mean EF percentile and self-report AQ scores for males and females

In order to reduce the number of comparisons, the associations between EF and ASD traits were examined using only the Mean EF score. Higher Mean EF scores were associated with marginally significantly higher scores on the AQ. When correlations were calculated for males and females separately, the correlation between Mean EF and AQ score was significant for females only. Fisher’s r test showed that the difference between these correlations did not reach statistical significance (z = −1.64, one-tailed p = .051), see Table 5 and Fig. 2.

**Table 5:** Correlations with between Mean EF Percentile and Self-report ASD traits and mood scales

	Whole sample	Male	Female
AQ (n = 133)	r = .160, p = .066	r = .062, p = .548	**r** = .**371, p** = .**024**^a,b^
EQ (n = 133)	**r** = **− .187, p** = .**031**^a^	r = **−** .160, p = .119	r = **−** .249, p = .137
SQ (n = 132)	**r** = .**280, p** = .**001**^a^	**r** = **235., p** = .**022**^a^	**r** = .**410, p** = .**012**^a^
BDI (n = 73)	**r = − .261, p** = .**026**^a^	r = **−** .265, p = .060	r = **−** .302, p = .172
BAI (n = 73)	r = **−** .221, p = .060	r = **−** .207, p = .146	r = **−** .393, p = .071

Significant results are given in bold

^a^Remains significant after controlling for age

^b^Fisher’s r comparing correlations for males versus females (z = **−** 1.64, two-tailed p = .101; one-tailed p = .051)

Higher Mean EF scores were also associated with significantly lower scores on the self-report EQ, and significantly higher SQ scores, see Table 5 for details. When correlations were explored by gender, neither correlation reached significance but both showed the same pattern of association and were not significantly different to each other.

Omitting the four older adults and repeating these analyses produced the same pattern of results (results not shown).

In the whole sample, the correlations were repeated controlling for the potentially covarying variables of age and depression. For SQ correlations with Mean EF associations controlling for age and depression remained significant (controlling for age: r = .29, p = .015; controlling for depression: r = .34, p = .003). Note that when controlling for depression the sample size is reduced (n = 73).

For EQ, the correlation with Mean EF remained significant after controlling for age (r = **−** .186, p. 033). However, when depression was included as a covariate in the Mean EF–EQ analysis, the correlation no longer reached significance (r = **−** .17, p = .147).

### Regression Analysis Examining Variables Associated with Mean EF Scores

Stepwise regression analysis was performed to investigate which variables explained variance in mean EF scores among adults receiving an ASD diagnosis. Included as independent variables were age, education level, AQ, EQ and SQ. The model significantly explained 14.7% of the variance in mean EF [F(1,109 = 18.84, p < .001)] with only educational level contributing to the model. A proportion of individuals completed mood questionnaires, therefore the regression was repeated including BDI and BAI. The model significantly explained 28.3% of the variance in mean EF scores [F(2,56) = 11.04, p < .001)]. Education level (22.6%) and BDI (5.7%), contributed significantly to explaining the variance.

### Regression Analysis Examining Variables Associated with Self-report AQ Scores

Stepwise regression analysis was performed to investigate which variables explained variance in AQ scores among adults receiving an ASD diagnosis. In both the models described below AQ score was the dependent variable.

#### Model 1

Included as independent variables in step 1 using stepwise function were the eight EF measures (Digit Symbol, Digit Span, Hayling, Brixton, Trails A and B, Zoo Map and Key Search), age and gender; EQ and SQ were included in a stepwise manner in step 2 (due to the expected high correlations between AQ, EQ and SQ).

The model significantly explained 48.8% of the variance in AQ scores (F(4,27) = 30.26, p < .001). Digit Symbol (8.8%), Digit Span (4.8%), EQ (27.9%) and SQ (7.2%), contributed significantly to explaining the variance.

#### Model 2

The above regression did not include mood scale information due to the reduced number of participants who completed these measures. The regression described in Model 1 was repeated with BDI and BAI included as independent variables in Step 1, with the regression otherwise repeated as above.

The model significantly explained 53.9% of the variance in AQ scores (F(5,66) = 15.43, p < .001). Zoo Map (6.7%), BAI (6%), Key Search (6.2%), EQ (26.3%) and SQ (8.7%) contributed significantly to the model.

Including non-linear effects in the models did not alter the results of the regression analyses. Results are not shown.

## Discussion

This study explored executive function abilities and autism traits in adults receiving a first diagnosis of ASD. As would be expected in a sample of individuals with cognitive abilities in the normal range, mean scaled scores for each executive function measure were in the average range. For all executive function measures, age-corrected scores (from normative data) were included in the analysis; if individuals with ASD show the same age-related trajectories as typical adults slopes would be expected to be flat and no significant correlations observed. For most measures, no significant correlations were noted between age and task performance in keeping with trajectories across the lifespan being the same in ASD and typical adults (for Digit Span, Brixton, Hayling, and Key Search tests). However on several measures requiring processing speed and (reactive flexibility) sequencing of performance (Digit Symbol, Trails A and B), correlations with age demonstrated a significant positive correlation, indicating that performance was better in ASD adults than in typical adults as age increased. As this study was exploratory in nature, further studies are required to replicate the results.

The positive association between performance on reactive flexibility measures and age among ASD adults with ability in the normal range, contrasts with results from other studies. In a sample with a similar age range also using a Trails test, Powell et al. (2017) found that age, IQ and sequencing ability predicted reactive flexibility in ASD. Powell et al.’s results suggest that older age was associated with greater difficulties in reactive flexibility. One other study using a different measure of reactive flexibility/reactive control (Simons task), found similar age-associations across the lifespan for ASD and non-ASD adults (Lever et al. 2017). Thus far in the three studies measuring reactive flexibility results are inconsistent, perhaps due to differences in sample characteristics or the tasks used. Further research is required to establish the pattern of age-related change in reactive flexibility across adulthood in ASD. It is interesting to note that the EF measures showing better than expected performance with age were timed measures that may also rely on information processing speed. Although information processing speed reduces in typical ageing (Salthouse 1993) studies of children with ASD suggest that processing speed difficulties may be due to motor difficulties rather than pure processing speed (Wallace et al. 2009; Oliveras-Rentas et al. 2012; Kenworthy et al. 2013). Associations with age in adults with ASD are not known. However, in one study that examined associations between processing speed and executive performance (spontaneous flexibility, fluency tasks; planning, Tower of London and Zoo Map) in a sample of ASD adults aged over 50, no significant correlations were observed (Davids et al. 2016). [Note, age-processing speed associations were not reported]. These results may be related to the specific domain of executive function being examined, but further exploration of the relationship between executive function and processing speed among older adults with ASD are required.

In the current study a measure of planning (Zoo Map) demonstrated a negative correlation with age, suggesting poorer performance than typical adults as age increased. The negative association with age was significant when excluding the four adults aged over 60, although did not reach significance in the whole sample (p = .078). The correlation may indicate a steeper decline in this function across adulthood in ASD compared to typical adults, although this difference may occur in midlife rather than later-life. This finding of planning showing a steeper age-related slope in ASD compared to typical adults, differs from two previous studies which found no significant association with age on planning measures (Towers tests, Davids et al. 2016; Geurts and Vissers 2012). As the current study is exploratory, caution should be applied when interpreting results that are not consistent across different analyses within the sample. It is unclear to what extent the difference in findings reflects the measures used, cohort differences or other explanations. Whereas Towers Tasks often include either a time-limit or time bonuses, the Zoo Map task is not time-based; however it seems unlikely that limited time would improve performance and lead to this pattern of results. A potentially more likely explanation relates to the reported ecologically validity of the Zoo Map task, compared to other neuropsychological measures of planning (Wilson et al. 1998). The Zoo Map task has relatively little external structure and relies on the individual generating and executing a successful plan. Therefore this task may be a better reflection of real-world planning, and results suggest that this may be impaired in adults with ASD.

Age-associations with ASD traits were also explored. In the current study, age correlated significantly with scores on both the AQ and the SQ. Few studies to date have explored ASD traits using a wide age-range, or have examined whether self-reported traits increase with older age in the typical population. In a previous study by our group including a sub-set of the adults with ASD reported here (Happe et al. 2016), age correlated with AQ and SQ scores in an adult ASD group but correlations were not significant in a non-ASD group (who had been referred with suspected ASD but did not receive a diagnosis). In a different sample, AQ scores demonstrated significant linear and non-linear associations with age: AQ scores were higher among older individuals with ASD, but scores showed a peak in middle-age (Lever and Geurts 2018). In previous studies of typical older adults, we have examined ASD traits using the Broad Autism Phenotype Questionnaire (BAPQ; Hurley et al. 2007; Wallace et al. 2016). In this typical population sample, although over-sampled for the BAP, there was no significant correlation with age for total BAPQ or the behavioural regulation index scores. However age correlated significantly with scores on the metacognition index. Overall results suggest that ASD traits do not correlate strongly with age in the typical population. It is unclear whether the positive correlations between AQ scores and age in the current sample reflect higher levels of ASD traits in midlife and later-life adults with ASD or characteristics of the sample possibly reflecting increased likelihood of receiving an ASD diagnosis.

Associations between performance on a composite EF score, ASD traits and mood were explored and gender effects considered. Across the whole sample, higher Mean EF scores were associated with marginally significantly higher scores on the AQ. For females but not males the association between Mean EF score and AQ scores reached significance, although the difference between the correlations for males and females was not significant. As women with ASD often report expending an enormous effort to fit in, perhaps this association reflects those highly able females with ASD using strategies to mask their ASD traits, although this is speculative. Across the whole sample, Mean EF scores were associated with higher scores on the SQ. One suggestion for this result is that an association may be present between cognitive ability and extracting rules and order from situations. Rather counter-intuitively, higher EF scores were associated with lower self-report EQ and depression scores. Perhaps higher EF abilities enables insight into areas of difficulty, or enables better functioning or development of coping strategies therefore better mood. Although correlations are low, they are significant and remain significant after controlling for age (EQ: r = − .186, p. 033; Depression: r = − .239, p = .045).

In a final analysis, we used regression analyses to examine which variables explained the variance in AQ scores in this group of adults diagnosed with ASD. Although previous studies in children and adults have identified associations between ASD traits and executive functions (Brunsdon and Happe 2013; Happe and Ronald 2008; Kenworthy et al. 2009), this has not previously been examined across the adult lifespan. In a two-set stepwise model, variance in AQ was explained by executive function measures of Digit Symbol and Digit Span, with other ASD traits (EQ, SQ) contributing significantly to the model. Neither age nor gender contributed significantly to the model. A second regression analysis was performed in a subgroup of ASD adults who had completed self-report ratings of depression and anxiety. Executive function ability (measured by the Zoo Map and Key Search planning tasks) and anxiety ratings explained a significant proportion of the variance in AQ scores, with other ASD traits (EQ, SQ) also contributing to the model. These results show a similar pattern to the correlations (significant associations between ASD traits and executive function ability), but importantly neither age nor gender contribute to explaining the variance in ASD traits. Anxiety symptoms do contribute to explaining the variance in ASD traits along with executive function abilities, but note that this is within a sub-group of individuals diagnosed with ASD. In keeping with studies in children with ASD, significant associations are observed between aspects of executive function and ASD traits (Brunsdon and Happe 2013; Happe and Ronald 2008; Kenworthy et al. 2009). Future studies may benefit from examining different aspects of ASD traits such as repetitive behaviours and their associations with different aspects of executive function.

There are some limitations to the current study. The individuals described here do not reflect the whole spectrum of ASD, as no-one included had an intellectual impairment and all had intelligence within the normal range. Executive function ability was compared to age-normative data rather than to a matched control group, although normative data provides a comprehensive population for comparison a direct comparison to a control group was not possible in this study. It is important to note that although the correlations discussed are significant, they are low to moderate. However, analyses are based on age-corrected scores, therefore results are significant after controlling for expected age-effects. Data on current medication use is unavailable and may have influenced performance on the executive function measures. Although all individuals received a first diagnosis of ASD in adulthood through a specialist clinical diagnostic unit we do not have access to a standardised assessment level such as the Autism Diagnostic Observation Schedule. However, self-report AQ scores were available and were in a similar range to those reported by other studies (this study, mean AQ = 34.75; Wheelwright et al. 2006, ASD mean AQ = 36.5, typical adults mean AQ = 16.3, 2010, parentsof ASD children mean AQ = 17.8; Ruzich et al. 2016, narrow autism phenotype mean AQ = 35). Late diagnosis may be an indicator of important differences from early diagnosed individuals with ASD, either due to presentation, intellectual ability, access to diagnostic services, coping with or masking ASD traits. Although all individuals received a diagnosis in adulthood the age-range reported here was wide with few individuals aged over 60 years of age. However, this group has not previously been widely studied and allows the opportunity to examine a group of adults, across a wide age-range who were all diagnosed according to the same clinical criteria (rather than age confounding with diagnostic criteria). An advantage of this sample is that it comprises approximately 28% females with ASD, on whom data is currently lacking. As with all cross-sectional studies, a limitation is that only association with age can be explored and longitudinal studies are required to investigate change. It is worth noting that this study relies on a convenience sample recruited through a diagnostic clinic and is exploratory in nature, therefore there is a risk that effects may be over-estimated. Results should be interpreted with caution and replication studies are required to fully understand the trajectory of executive function in ageing with ASD.

It is not yet clear if the pattern of positive correlations in reactive flexibility with age reported here represents real “protection” for ASD adults across the lifespan, or is related to some sample-specific features of the group being described or the exploratory nature of the analyses. There are several ways of viewing a potentially “protective” factor. For example, it is possible that across the lifespan individuals with ASD have regularly utilised executive control strategies in order to cope with the demands of daily life; therefore their executive control abilities may be honed to perform at a high level. A similar suggestion of lifelong executive control effort resulting in improved performance in late adulthood has been made in the typical ageing literature, particularly with reference to bilingual older adults (Bialystok et al. 2012). It is worth noting that age-scaled scores in the ASD adults reported here are in the average range. A related but alternative explanation is that individuals with ASD may already have strategies in place, which allow them to cope with the usual decline in cognitive performance that occurs across the adult lifespan, whereas typical older adults may be only beginning to develop these strategies (if indeed they develop them at all), once decline in function has commenced. Alternatively, this may not represent an “improvement” with age, but rather a less steep decline in abilities or a “maintenance” of abilities across adulthood in ASD.

In summary, in a group of adults without intellectual disability receiving a first ASD diagnosis in adulthood, few gender differences in executive function were observed. In some but not all executive function tasks, significant correlations were observed between age and task performance. Results suggest that for some executive function abilities relying on speed and sequencing, late-diagnosed individuals with ASD may demonstrate better age-related performance than typical age-matched peers. However, a trend towards poorer planning ability in age-related performance was also observed. Results suggest that different aspects of executive function may show differential trajectories across the adult lifespan in ASD compared to typical adults; with patterns of slower, accelerated or equivalent age-related associations all being observed. Although cross-sectional results can inform research on lifespan trajectories of ASD, longitudinal studies are required to fully investigate the pattern of age-related change.
